# The Catalytic Roles of P185 and T188 and Substrate-Binding Loop Flexibility in 3α-Hydroxysteroid Dehydrogenase/Carbonyl Reductase from *Comamonas testosteroni*

**DOI:** 10.1371/journal.pone.0063594

**Published:** 2013-05-23

**Authors:** Chi-Ching Hwang, Yi-Hsun Chang, Hwei-Jen Lee, Tzu-Pin Wang, Yu-Mei Su, Hsin-Wei Chen, Po-Huang Liang

**Affiliations:** 1 Department of Biochemistry, Faculty of Medicine, College of Medicine, Kaohsiung Medical University, Kaohsiung, Taiwan; 2 Department and Graduate Institute of Biochemistry, National Defense Medical Center, Taipei, Taiwan; 3 Department of Medicinal and Applied Chemistry, Kaohsiung Medical University, Kaohsiung, Taiwan; 4 Institute of Biological Chemistry, Academia Sinica, Taipei, Taiwan; Russian Academy of Sciences, Institute for Biological Instrumentation, Russian Federation

## Abstract

3α-Hydroxysteroid dehydrogenase/carbonyl reductase from *Comamonas testosteroni* reversibly catalyzes the oxidation of androsterone with NAD^+^ to form androstanedione and NADH. Structurally the substrate-binding loop of the residues, T188-K208, is unresolved, while binding with NAD^+^ causes the appearance of T188-P191 in the binary complex. This study determines the functional roles of the flexible substrate-binding loop in conformational changes and enzyme catalysis. A stopped-flow study reveals that the rate-limiting step in the reaction is the release of the NADH. The mutation at P185 in the hinge region and T188 in the loop causes a significant increase in the *K_d_* value for NADH by fluorescence titration. A kinetic study of the mutants of P185A, P185G, T188A and T188S shows an increase in *k_cat_, K_androsterone_* and *K_iNAD_* and equal primary isotope effects of *^D^V* and *^D^*(*V/K*). Therefore, these mutants increase the dissociation of the nucleotide cofactor, thereby increasing the rate of release of the product and producing the rate-limiting step in the hydride transfer. Simulated molecular modeling gives results that are consistent with the conformational change in the substrate-binding loop after NAD^+^ binding. These results indicate that P185, T188 and the flexible substrate-binding loop are involved in binding with the nucleotide cofactor and with androsterone and are also involved in catalysis.

## Introduction

**Figure 1:**
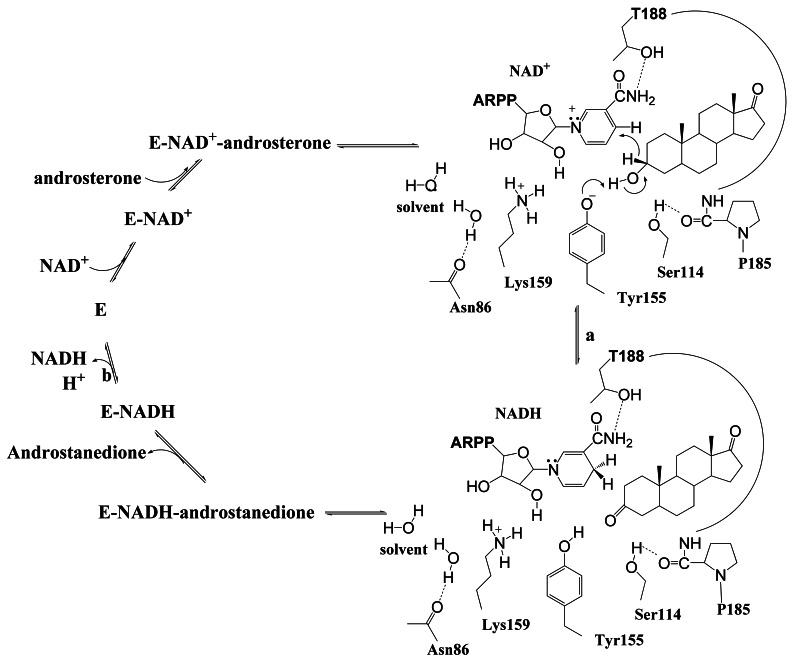
The mechanism of the 3α-hydroxysteroid dehydrogenase/carbonyl reductase-catalyzed reaction. 3α-HSD/CR reversibly catalyzes the oxidation of androsterone with NAD^+^ to form androstanedione with NADH in a sequential order bi bi kinetic mechanism with NAD^+^ added first and NADH released last. (a) The chemical step wherein a tetrad of catalytically important N86, S114, Y155 and K159 residues in the active site are shown. Y155 acts as a general base to facilitate the hydride transfer from the 3β-hydrogen of androsterone to the nicotinamide ring. S114 interacting with P185 maintains the conformation of the substrate-binding loop, in which T188 binds with NAD^+^. (b) The rate-limiting step for NADH and proton release. Protons are released through the proton relay system, via Y155, K159 and N86, to the solvent.

3α-Hydroxysteroid dehydrogenase/carbonyl reductase (3α-HSD/CR; EC1.1.1.50) from *Comamonas testosteroni*, reversibly catalyzes the oxidation of androsterone with NAD^+^ to form androstanedione and NADH. Its expression in *C. testosteroni* is induced when testosterone and progesterone are present and it is involved in the initial degradation of steroids to provide carbon and energy [1], [2]. The reaction catalyzed by 3α-HSD/CR shows an ordered bi bi kinetic mechanism, in which NAD^+^ and then androsterone binds with the enzyme to form a ternary complex. The products, androstanedione and NADH, are then released sequentially after the oxidoreduction reaction [3]. Studies of steady-state kinetic and solvent isotope effects indicate that the release of the NADH product coupled with proton transfer is the rate-limiting step in the overall reaction that is catalyzed by 3α-HSD/CR [4]. 3α-HSD/CR belongs to the short chain dehydrogenase/reductase (SDR) superfamily [5]–[8]. The SDR enzymes are 250–350 residues in length. Most SDR enzymes utilize NAD(H) or NADP(H) as a cofactor and are involved in the inter-conversion of the active hormone and its inactive metabolite [9], [10]. Structurally, SDR enzymes have an α/β folding pattern, similar to a Rossmann fold, which consists of a central β-sheet that is flanked by α-helices, while NAD(P)^+^ is bound at the C-termini of the parallel β-strands [1], [11], [12]. The alignment of sequences between different SDR enzymes shows conserved sequences, including a tetrad of catalytically important Ser, Tyr, Lys and Asn residues, and an N-terminal Gly-X_3_-Gly-X-Gly cofactor binding motif [13]. The function of the catalytic tetrad N86-S114-Y155-K159 in 3α-HSD/CR catalyzed reaction has been characterized in detail (Figure 1) [4], [7], [11], [14]–[16]. Y155 is involved in the acid-base catalysis to facilitate the hydride transfer from the 3β-hydrogen of androsterone to the nicotinamide ring, where a proton transfer from the hydroxyl group of Y155, via the 2′-OH of the nicotinamide ribose, the K159 side chain and a water molecule hydrogen-bonded to the backbone carbonyl of N86 constitutes a proton relay system.

In many SDR enzymes, the substrate-binding loop is involved in the recognition of substrate structure and demonstrates an intrinsic flexibility. It is almost completely disordered in the absence of a bound cofactor and substrate, but becomes ordered after substrate binding [17]–[21]. The ligand-binding induced motion of the substrate-binding loop has been demonstrated to guide the enzyme along its preferred catalytic pathway [22], [23]. The motion of the loop is involved in binding with the ligands, which facilitates a reaction by an optimal interaction with the transition state, followed by the release of the products [24], [25]. It plays a functional role via its motion between the two major conformational states, the open and closed conformations. The ligands can access both the active site and the solvent in the open conformation and are bound in the active site in the closed conformation to shield the enzyme-substrate interaction from the solvent.

**Figure 2:**
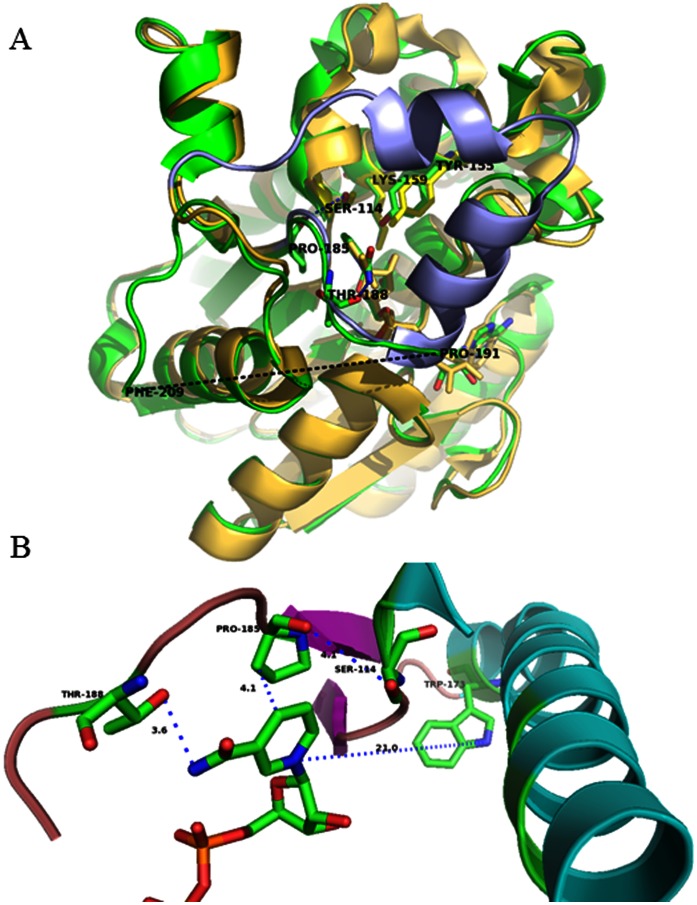
Structural alignment of *P.s.* 3α-hydroxysteroid dehydrogenase and *C.t.* 3α-HSD/CR. A. Coenzyme NADH binding to *P.s.* 3α-HSD (yellow; pdb:2dkn) induces the loop-helix transition (blue) and an unresolved flexible loop (black dotted line) between P191 and F209 for the NAD^+^ bound *C.t.* 3α-HSD/CR (green; pdb:1fk8). NAD^+^ and key residues in the active site of 3α-HSD/CR are also labeled. (B) Close-up of the interaction of the substrate-binding loop with NAD^+^. The interactions of P185 at the hinge region with both S114 and nicotinamide ring, and T188 in the substrate-binding loop with the amide NH of NAD^+^ are shown as blue dotted lines. The distances from P185 to the nicotinamide ring of NAD^+^ and the hydroxyl group of T188 with the amide NH of NAD^+^ are 4.1 and 3.6 Å, respectively. The image was generated using the PyMOL program.

The crystal structures of apo- and NAD^+^-bound 3α-HSD/CR have been determined and exhibit an incomplete and unresolved substrate-binding loop of T188-K208 for the apoenzyme and L192-K208 for the NAD^+^-bound complex, respectively [11], [14]. A significant induced conformational change from a disordered substrate-binding loop to a closed helix-turn-helix is demonstrated in the binding of NADH with *Pseudomonas sp.* 3α-HSD (17). The binding of NADH with *C.t.* 3α-HSD/CR causes the appearance of T188-P191 in the substrate-binding loop, where T188 is involved in the interaction with the nicotinamide of NAD^+^ (Figure 2). It is proposed that S114 is important in maintaining the conformation of the substrate-binding loop to allow nucleotide cofactor binding, which facilitates the reaction [14]. However there is no detailed study of the relationship between the conformational flexibility of the substrate-binding loop and binding and catalysis. This study determines the functional role of the substrate-binding loop in the 3α-HSD/CR from *C. testosteroni* and the particular roles of the loop residues, P185 and T188. Using site-directed mutagenesis, spectrophotometric analysis, kinetic study and molecular simulations, the role of the flexibility of the substrate-binding loop that is involved in the enzyme function is studied. An attempt is made to increase the flexibility of the substrate-binding loop via mutations at P185 in the hinge region in order to decrease the backbone hydrogen-bonding constraints, and via mutations at T188 in order to interrupt the interaction with NAD^+^ and probe its role in the conformational changes and in catalysis. The mutants of P185A, P185G, T188A, T188S and the double mutants of W173F/P185W and W173F/T188W 3α-HSD/CRs are constructed, expressed, purified and characterized. The results demonstrate that a balance of structural rigidity and flexibility in the substrate-binding loop is essential for enzyme catalysis.

## Materials and Methods

### Materials

A QuikChange Site-Directed Mutagenesis kit from Agilent Technologies was used. Androsterone and androstanedione were purchased from Steraloids, Inc. and NAD^+^, NADH, glucose-6-phosphate dehydrogenase from *Leuconostoc mesenteroids* were purchased from Sigma. The deuterated chemicals, 1-*deutero*-glucose (95%-^2^H), came from Cambridge Isotope Laboratories. All chemicals were of the highest purity available.

### Expression and Purification of Wild-type and Mutant Enzymes

Mutagenic replacements were performed using a QuikChange site-directed mutagenesis kit with Pfu polymerase and pet15b-3α-HSD/CR plasmid as the template [15]. The primers used to create the cDNA encoding for the P185A, P185G, P185W, T188A, T188S and T188W mutants, and the double mutants of W173F/P185W and W173F/T188W are shown in Table S1. The mutant vectors were transformed into competent *Escherichia coli* BL21(DE3) cells. All mutants of 3α-HSD/CR cDNA were sequenced to ensure fidelity. The overexpressed proteins were purified as wild-type recombinant 3α-HSD/CR and prepared as described previously [15]. In brief, recombinant proteins were overexpressed in BL21 (DE3) cells and grown at 37°C to an optical density of 0.6–1 at 600 nm in a LB medium containing 50 µg/ml ampicillin. Isopropyl β-D-thiogalactopyranoside (0.5 mM) was added to the culture to induce protein expression, and growth was continued for an additional 4 h at 37°C. The cells were then harvested and lysed by sonication. The lysate was loaded into a Ni^2+^-nitrilotriacetic acid affinity column and eluted via a stepwise increase in the concentration of imidazole from 20 mM to 500 mM to purify the protein. These enzymes were purified to homogeneity, as determined using SDS-PAGE. The protein concentrations were determined by a Bradford assay, using bovine serum albumin as a standard [26].

### Circular Dichroism Measurements

The secondary structures of the wild-type and mutant 3α-HSD/CRs, the E-NADH binary complex and the E-NADH-androsterone ternary complex in solution were assessed using circular dichroism (CD) spectroscopy by measuring the ellipticity using a 0.02 mm pathlength cuvette in the range 190–250 nm at a resolution of 1 nm with a scanning speed of 20 nm/min and a 1 s response time at room temperature with a JASCO J-810 spectropolarimeter. Spectra were obtained as the average of three consecutive spectra of 8.8 µM enzymes in the absence and in the presence of 100 µM NADH and 50 µM androsterone at 10 mM phosphate at pH 7.5.

### Fluorescence Measurement

Fluorescence measurements were performed on a LS55 Luminescence spectrometer (Perkin Elmer) at room temperature. The excitation wavelength used was 295 nm and the emission range was recorded from 300 to 500 nm at room temperature. The slit width for both emission and excitation was 3.5 nm. The wild-type and mutant enzymes were measured at a final concentration of 2 µM in 40 mM Hepes at pH 7.5. The spectra were corrected for background by subtracting the buffer spectra. The binding of NADH with mutant enzymes was measured by quenching the intrinsic enzyme fluorescence at maximum wavelength after the incremental addition of NADH. The fluorescence intensity from 300 to 500 nm was measured in a solution of 2 µM enzyme and varied concentrations of NADH in 40 mM Hepes at pH 7.5. The decreased intensity of 3α-HSD/CR’s fluorescence emission at maximum wavelength after the addition of NADH was corrected for an inner filter effect caused by the absorbance of NADH at 295 nm [27].

### Steady-state Kinetics Studies

The oxidation of androsterone catalyzed by 3α-HSD/CR was measured by the formation of NADH, using a spectrophotometer at 340 nm. The initial rate pattern was obtained by varying the concentration of androsterone at several fixed concentrations of NAD^+^ in 0.1 mg/mL BSA, 0.1 M Caps at pH 10.5 at 25°C. All reactions were initiated by the addition of an enzyme. The primary kinetic isotope effects, *^D^V_max_* and *^D^*(*V_max_/K_androsterone_*), were determined by direct comparison of the kinetic parameters, *V_max_* and *V_max_/K_androsterone_,* with undeuterated and deuterated androsterone. [3α-^2^H]Androsterone was prepared by enzymatic synthesis from [4S-^2^H]NADH with 5α-androstan-3,17-dione catalyzed by 3α-HSD/CR as described previously [4]. The values of *V_max_* and *V_max_/K_androsterone_* were obtained by measuring the initial rate as a function of either the deuterated or the undeuterated androsterone concentration at 1.7 mM NAD^+^. The concentrations of deuterated and undeuterated androsterone were determined three times by an end point assay.

### Stopped-flow Experiments

Stopped-flow measurements were performed using an Applied Photophysics SX-18 MV stopped-flow spectrofluorimeter (Leatherhead, Surrey, UK). The stopped-flow experiments were performed by mixing wild-type 3α-HSD/CR that was preincubated with NAD^+^ with equal volumes of varied concentrations of androsterone in 0.1 M Caps at pH 10.5. The NADH fluorescence emission above 420 nm was measured using an excitation wavelength of 340 nm. A cut-off filter was used to prevent the detection of any fluorescence signal below 420 nm. The observation cell with a 1 cm pathlength was maintained at 25°C during the stopped-flow measurements. The apparent observed rate was obtained by fitting the stopped-flow trace with a single exponential equation.

### Structural Simulation

The modeled structure of the binary complex of 3α-HSD/CR was generated using a build homology models protocol (Accelrys Discovery Studio 3.1, Accelrys Inc.). The structures of 3α-HSD/CR from *C. testosteroni* (PDB: 1FK8) and *Pseudomonas* sp. B-0831 (PDB: 2DKN) were used as templates. The sequences of the template and the target were aligned using the protocol, Align Sequence to Templates. The ligand of NAD^+^ from the binary complex of 3α-HSD/CR was copied to the model in the simulation. A forcefield of CHARMm was typed for the simulation. Loop refinement was included in the build. The default settings of other parameters were used. The quality of the model was verified by the protocol, Verify Protein (Profiles-3D), and judged to be satisfactory. Energy minimization was applied to the model using the protocol of Minimization with the algorithm of smart minimization and including the implicit solvent model of Generalized Born in the calculation until the gradient tolerance was satisfied (RMS Gradient ∼0.1 kcal/mol/Å). The modeling of 3α-HSD/CR used the same protocol but a different template structure of 3α-HSD/CR (PDB: 1FJH). The loop in the resulting structural model (T188 to K208) was refined using the protocol, Loop Refinement. The conformation with the highest ranking was then used for energy minimization.

### Data Analysis

The data were fitted using Sigmaplot software for appropriate rate equations to obtain the kinetic parameters. The data for the substrate saturation curves at a fixed concentration of the second substrate were fitted using Equation 1, where *v* and *V* represent the initial and maximum velocities, respectively, and *K_A_* is the Michaelis constant for substrate A. Double reciprocal plots of the families of lines, in which the initial velocities are plotted against substrate concentrations were constructed [28]. The data fitted to the rate equation for either a sequential or a rapid equilibrium order kinetic mechanism were fitted to Equations 2 and 3, respectively, where A and B are the varied substrates, *K_ia_* is the inhibition constant for A and *K_A_* and *K_B_* are the Michaelis constants for substrates A and B, respectively. The effect of mutation on the binding interactions between the enzyme and the cofactor or the transition state was determined using Equations 4 and 5, respectively [29]. *R* is the gas constant (1.987 cal mol^−1^ K^−1^) and *T* is the temperature in Kelvin (298 K). The data from the experiments for the isotope effects were fitted to Equations 6–8, when an isotope effect was observed on both *V* and *V/K*, an equal isotope effect on *V* and *V/K*, and *V/K* only, respectively. In Equations 6–8, *F_i_* is the fraction of the deuterium label in the substrate, *E_V_,_V/K_* is the isotope effect minus 1 for an equal isotope effect on *V* and *V/K*, and *E_V_* and *E_V/K_* are the isotope effect minus 1 on *V* and *V/K*, respectively. The data for the burst kinetics, in which an exponential increase in fluorescence (F_t_) is followed by a linear steady state increase in F_t_, were fitted to Equation 9: ∏ is the burst constant, *k_obs_* (s^−1^) and *k_ss_* are rate constants. The dissociation constant (*K_d_*) for NADH was determined by fluorescence titration. The correction for inner filter effect was done by measuring the increased absorbance at 295 nm in the presence of increasing concentrations of NADH in the same buffer. The correction was obtained by fitting to Equation 10, where *F*, *I_0_* and *I_obs_* are the correction factor and the corrected and observed fluorescence intensity, respectively. *P_0_* and *ΔA* are the sample absorbance at 295 nm before titration and the absorbance change with addition of ligand, respectively. After correction for the inner filter effect, the difference in enzyme fluorescence (*ΔF*) from titration with NADH was fitted to Equation 11, where *F_0_* and *F* are the corrected fluorescence intensities in the absence and presence of ligand (*L*), respectively, and *F_max_* and *K_d_* are the maximum fluorescence and dissociation constant for the ligand, respectively.
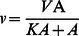
(1)

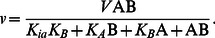
(2)

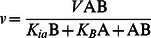
(3)

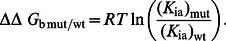
(4)

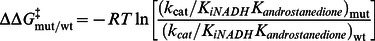
(5)

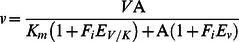
(6)

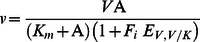
(7)

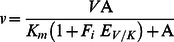
(8)

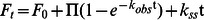
(9)

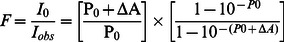
(10)

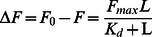
(11)


## Results

### Structural Probe of the Mutant 3α-HSD/CRs

**Figure 3:**
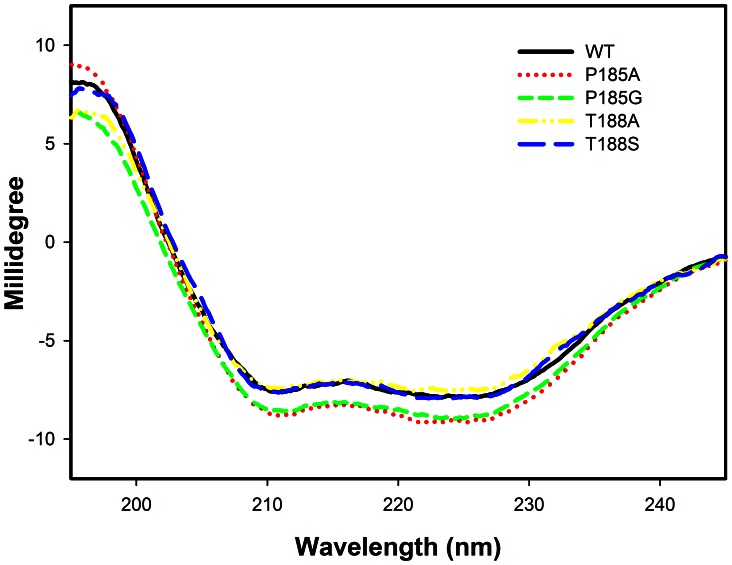
CD spectra of wild-type 3α-HSD/CR and mutant enzymes. The CD spectra of wild-type and the P185A, P185G, T188A and T188S mutant enzymes. The mutation at P185 causes an increase in the intensity at 222 nm, compared to that of the wild-type enzyme. The CD spectra were measured at 8.8 µM enzyme in 10 mM phosphate at pH 7.5 at room temperature.

**Figure 4:**
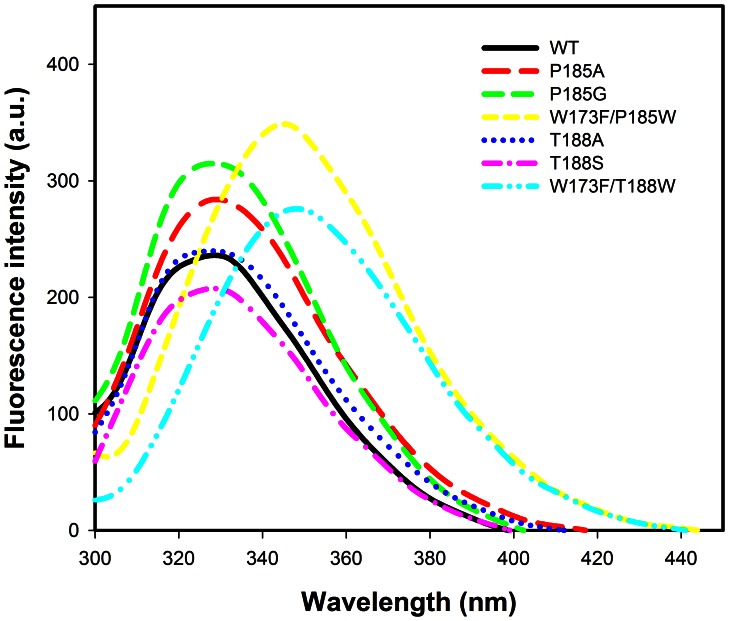
The fluorescence emission spectra of wild-type 3α-HSD/CR and its mutants. The fluorescence spectra of wild-type and P185A, P185G, T188A, T188S, W173F/P185W, and W173F/T188W mutant enzymes. The mutants of W173F/P185W and W173F/T188W display a red shift in the maximum wavelength at 345 and 349 nm, respectively. The protein fluorescence spectra were measured at 2 µM enzyme in 40 mM Hepes at pH 7.5 at room temperature. The excitation wavelength is 295 nm and the emission range was recorded from 300 to 450 nm.

The secondary structures of the wild-type and mutants of P185A, P185G, T188A and T188S 3α-HSD/CRs were assessed by CD spectroscopy by measuring the ellipticity in the 190–250 nm range at room temperature. No difference in the CD spectra was observed for the T188 mutants, but an increase in the intensity at 222 nm was observed for P185 mutants, compared to that of the wild-type enzyme, which indicates an increase in the α-helix (Figure 3). The addition of 100 µM NADH to the P185 and T188 mutated enzymes to form E-NADH binary complex and the further addition of 50 µM androsterone to form E-NADH-androsterone ternary complex has a CD spectrum similar to that of the apoenzyme, which suggests that there is no further change in the secondary structure of mutants induced by the ligands. In order to determine whether the conformation of 3α-HSD/CR is affected by the mutation at the substrate-binding loop, the fluorescence spectrum for the apo- and binary complex of wild-type and mutant enzymes was measured. Tryptophan residue is a useful fluorescence probe as the indole ring is sensitive to its environment. 3α-HSD/CR has a single tryptophan residue (W173) located at the C-terminus of the α-helix F, which contributes to the observed intrinsic tryptophan fluorescence signal when excited at 295 nm. The fluorescence spectra for the wild-type and mutant 3α-HSD/CRs are shown in Figure 4. The mutants of P185A, P185G, T188A and T188S have a spectrum similar to that of the wild-type enzyme with a maximum wavelength at 329 nm. The fluorescence intensity is in the order: P185G>P185A>T188A∼WT>T188S. In order to identify the local environment of the substrate-binding loop, the double mutants of W173F/P185W and W173F/T188W with a single tryptophan residue placed in the substrate-binding loop were constructed. The maximum wavelengths for the mutants of W173F/P185W and W173F/T188W were shifted to 345 and 349 nm, respectively, which indicates the residues, W185 and W188, in the substrate-binding loop are exposure to water medium [30]. For comparison, the fluorescence spectrum for free tryptophan in aqueous solution and the unfolding wild-type enzyme at 6 M urea were measured and showed a maximum wavelength at 356 and 355 nm, respectively. Therefore, the red shift of the emission spectrum of the mutated enzyme suggests that the solvent exposes tryptophan residue. This result confirms that the substrate-binding loop is exposed to the solvent [31], [32].

### Fluorescence Quenching and Determination of the Binding Constants of the Wild-type and Mutant 3α-HSD/CRs for NADH

**Figure 5:**
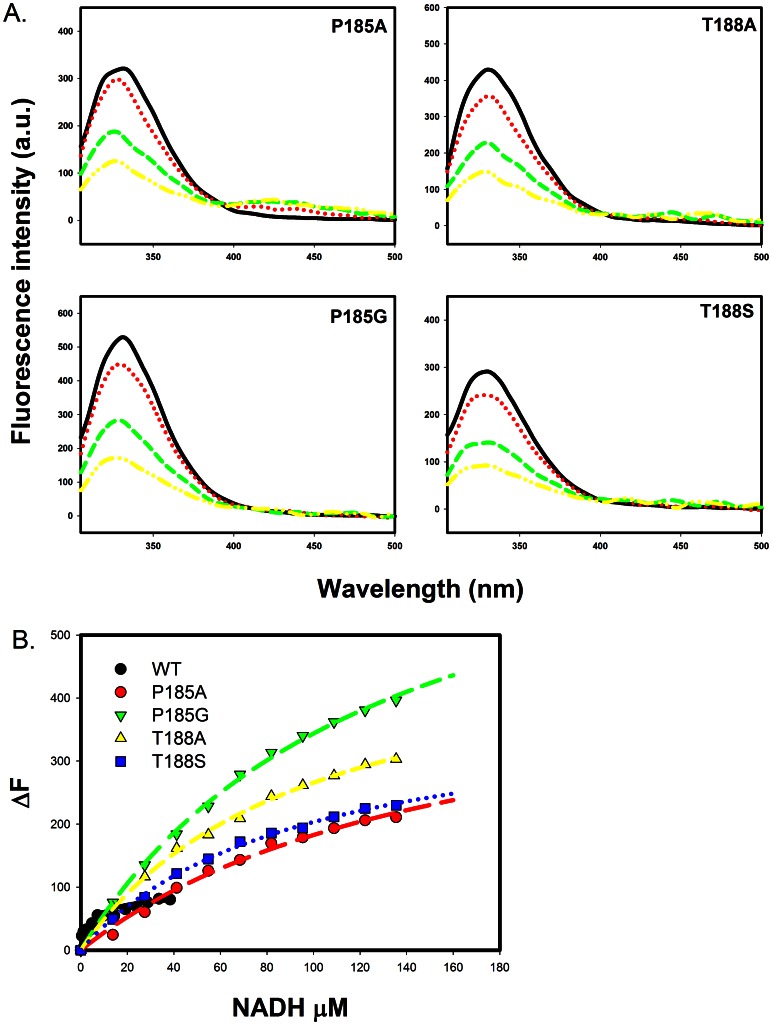
The fluorescence titration spectra of 3α-HSD/CR and its mutants. A. The fluorescence titration spectra of the mutant enzymes of P185A, P185G, T188A and T188S for increased addition of NADH. The incremental addition of NADH gradually causes a blue shift at the maximum wavelength for the P185A and P185G mutants. For clarity, only 0(black), 14(red), 55(green) and 97(yellow) µM NADH titrations are shown. B. The fluorescence titration curves by varying the concentrations of NADH for the wild-type and P185A, P185G, T188A and T188S mutant enzymes. Corrected for the inner filter effect, the difference (ΔF) in the intrinsic protein fluorescence titrated by NADH is shown. The lines represent the fit of the data points of the wild-type and mutants to Equation 11. The data for the wild-type enzyme is from Ref. 16.

The effect of conformational change on tryptophan accessibility and the binding constant for NADH were studied by protein fluorescence quenching by adding NADH at pH 7.5. Binding of NADH with wild-type and mutant enzymes causes a decrease in the fluorescence intensity at 329 nm when the tryptophan residue is excited at 295 nm. The fluorescence quenching of the mutant enzymes by NADH is shown in Figure 5. The binding of NADH with mutant enzymes quenches the intrinsic tryptophan emission intensity, but does not affect the maximum fluorescence wavelength for the T188A and T188S mutants. Incremental addition of NADH gradually causes a blue shift to 327 nm for the P185A and P185G mutants, which suggests a change to a buried residue for W173 when NADH is bound in the active site [30]. The dissociation constant (*K_d_*) for NADH with mutant enzymes was determined by measuring the decrease in the fluorescence intensity at the maximum wavelength when excited at 295 nm during titration with NADH. The fluorescence intensity was corrected for the inner filter effect that is caused by the NADH absorption at this wavelength by fitting to Equation 10. The data for the difference in the corrected protein fluorescence were then fitted to Equation 11 (Figure 5). The values obtained for *K_d_* are 163±29, 130±9, 97±6 and 94±6 µM, for the NADH-enzyme complexes of the P185A, P185G, T188A and T188S mutants, respectively. The value of *K_d_* for the wild-type NADH-enzyme complex is 2.8±0.9 µM [16], which represents a 30- to 60-fold increase in the value of *K_d_* for the mutant enzymes compared to that for the wild-type enzyme.

### The Steady-state Kinetics of the Mutant 3α-HSD/CRs

The steady-state kinetics of mutants of P185A, P185G, T188A and T188S 3α-HSD/CR catalyzed the oxidation of androsterone with NAD^+^ was studied by varying the concentration of androsterone at several fixed concentrations of NAD^+^ at pH 10.5. The double-reciprocal plots for the P185A and P185G mutant enzymes intersect to the left of the ordinate, which suggests a sequential kinetic mechanism (Figure S1). The data were fitted to Equation 2. While an intersection with the ordinate is observed for the T188A and T188S mutants, the data were fitted to Equation 3 for a rapid equilibrium sequential kinetic mechanism. The kinetic data for the mutant 3α-HSD/CRs are shown in Table 1. The activity is increased by 3- to 14-fold for the P185A, P185G, T188A and T188S mutant enzymes in *k_cat_*. The kinetic parameter, *k_cat_/K_androsterone_*, for the P185A mutant is increased 3-fold and is slightly decreased for the P185G, T188A and T188S mutants. A 17- to 83-fold increase in the inhibition constant (*K_iNAD_*) of NAD^+^ with mutants is observed.

**Table 1:** The kinetic constants and isotope effects for wild-type and mutant 3α-HSD/CR^a^.

	WT^b^	P185A	P185G	T188A	T188S
*k_cat_* (s^−1^)	100±8	916±61	273±10	1200±133	1414±149
*k_cat_/K_a_* (µM^−1^s^−1^)	8.2±0.1	4.7±1.1	1.3±0.4	NA	NA
*k_cat_/K_b_* (µM^−1^s^−1^)	26±3	83±12	19±3	23±3	22±3
*K_ia_* (mM)	0.06±0.03	1.0±0.3	5±1	1.4±0.2	1.6±0.3
*K_a_* (µM)	121±26	195±55	211±66	NA	NA
*K_b_* (µM)	3.8±0.8	11±2	15±3	52±11	64±16
*^D^V*	1	1.9±0.1	1.62±0.10	1.84±0.06	1.65±0.06
*^D^(V/K*_b_)	1.8±0.2	1.9±0.1	1.62±0.10	1.84±0.06	1.65±0.06

a The initial rate pattern was carried out by varying the concentrations of androsterone at different fixed concentrations of NAD^+^ in 0.1 M Caps at pH 10.5. *K_a_* and *K_b_* are the Michaelis constants of NAD^+^ and androsterone, respectively, while *K_ia_* is the inhibition constant of NAD^+^. NA, not available for rapid equilibrium order kinetic mechanism. The kinetic isotope effects were performed with varying concentrations of the unlabeled and labeled androsterone at 1.7 mM NAD^+^, pH 10.5.

b The kinetic parameters of wild-type enzyme are from Ref. 4.

### Stopped-flow Kinetic Study of the 3α-HSD/CRs

**Figure 6:**
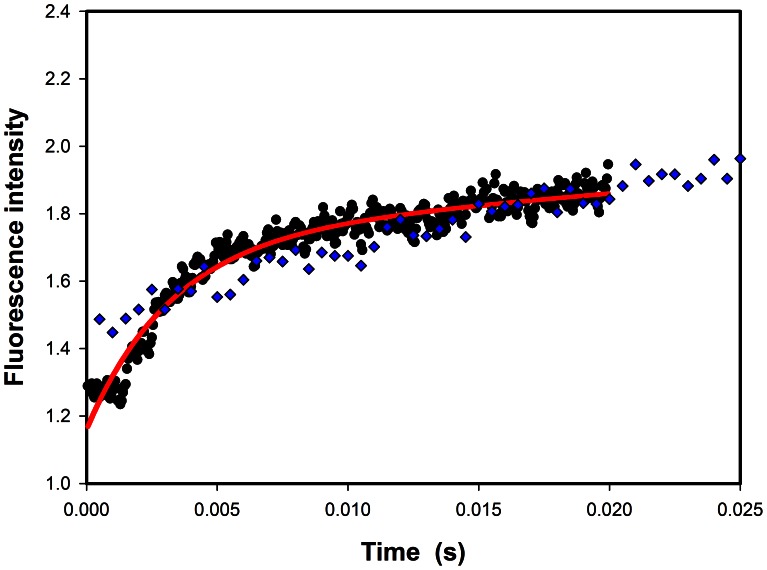
Pre-steady-state kinetics of 3α-HSD/CRs. The stopped-flow progress curves for 3α-HSD/CRs catalysis of the reaction of the undeuterated (blue diamond) and deuterated (black circle) androsterone with NAD^+^. Reactions were performed in 1 µM WT, 1 mM NAD^+^, 17.5 µM undeuterated or deuterated androsterone at 0.1 M Caps, pH 10.5. The line represents the fit of the data points to Equation 10, giving an apparent value for *k_obs_* of 309 s^−1^ for the reaction catalyzed by 3α-HSD/CRs with the deuterated androsterone.

In order to determine the rate-limiting step(s) in the 3α-HSD/CRs catalyzed reaction, the transient kinetics for the oxidation of an undeuterated and deuterated androsterone with NAD^+^ were studied. The stopped-flow experiment was performed in 1 µM enzyme with saturated NAD^+^ and 17.5 µM undeuterated or deuterated androsterone. A burst phase of the bound reduced nucleotide cofactor is observed on the deuterated androsterone but not on the undeuterated androsterone in the stopped-flow measurement (Figure 6). An observed burst phase indicates the rate-limiting step for the release of NADH and is consistent with the results from the kinetic primary isotope effect by the steady-state kinetics [4]. The observed kinetic traces for the deuterated androsterone were fitted to Equation 9, which gives an apparent rate constant of 309 s^−1^ for the deuteride transfer. This result indicates that the rate of catalysis is fast for undeuterated androsterone, so the burst phase of the bound NADH in the dead time (∼1.5 ms) within the time limit for stopped-flow measurement cannot be observed. Deuterated androsterone slows the chemical step for the hydride transfer, upon the appearance of the burst phase.

### Isotope Effect Studies

The rate-limiting step for the mutant enzyme catalysis of the oxidation of androsterone with NAD^+^ was further probed by determining the primary kinetic isotope effects on *V_max_* and *V_max_/K_androsterone_*. The reaction was performed using varying concentrations of undeuterated and deuterated androsterone at 1.7 mM NAD^+^, 0.1 M Caps, pH 10.5. The primary kinetic isotope effect was obtained by direct comparison of the kinetic parameters, *V_max_* and *V_max_/K_androsterone_*, for deuterated and undeuterated androsterone. The double-reciprocal plots for the wild-type and mutant enzymes are shown in Figure S2. The data show an equal isotope effect of around 1.8 on kinetic parameters, *V_max_* and *V_max_/K*_androsterone_, for the P185 and T188 mutant enzymes (Table 1).

### Changes in the Cofactor Binding Energy in the Mutants

The effects of mutation on the binding interactions between the enzyme and the nucleotide cofactor were studied for the P185A, P185G, T188A and T188S mutant 3α-HSD/CRs. The contributions to ground-state binding energy and transition-state binding energy were determined using Equations 4 and 5, respectively. The mutants of P185G, T188A and T188S destabilize both ground-state and the transition-state with a similar amount of binding energy (Table 2). The substitution of P185 for alanine causes greater destabilization in the ground-state than in the transition-state by 0.7 kcal/mol.

**Table 2:** Specificity constants and free energy differences in ground state and transition state energies between wild-type and mutant 3α-HSD/CRs^a^.

	WT	P185A	P185G	T188A	T188S
*k_cat_/K_ia_K_b_* (mM^−2^s^−1^)	42 ×10^4^	8.3×10^4^	0.40×10^4^	1.7×10^4^	1.4×10^4^
ΔΔG^‡^ *_mut/wt_* (kcal/mol)		0.96	2.8	1.9	2.0
ΔΔG*_bmut/wt_* (kcal/mol)		1.7	2.6	1.8	1.9

a The differential binding energy for the ground-state enzyme-NAD^+^ complex and the transition state of the the reaction catalyzed by the wild-type and mutant 3α-HSD/CRs at pH 10.5 were calculated using Equation 4 and 5, respectively. ΔΔ*G_b mut/wt_* and ΔΔ*G* ^‡^ *_mut/wt_* are the differential ground- and transition-state binding energies, respectively. *k_cat_/K_ia_K_b_* is referred to *k_cat_/K_iNAD_K_androsterone_* obtained from Table 1.

### Homology Modeling

**Figure 7:**
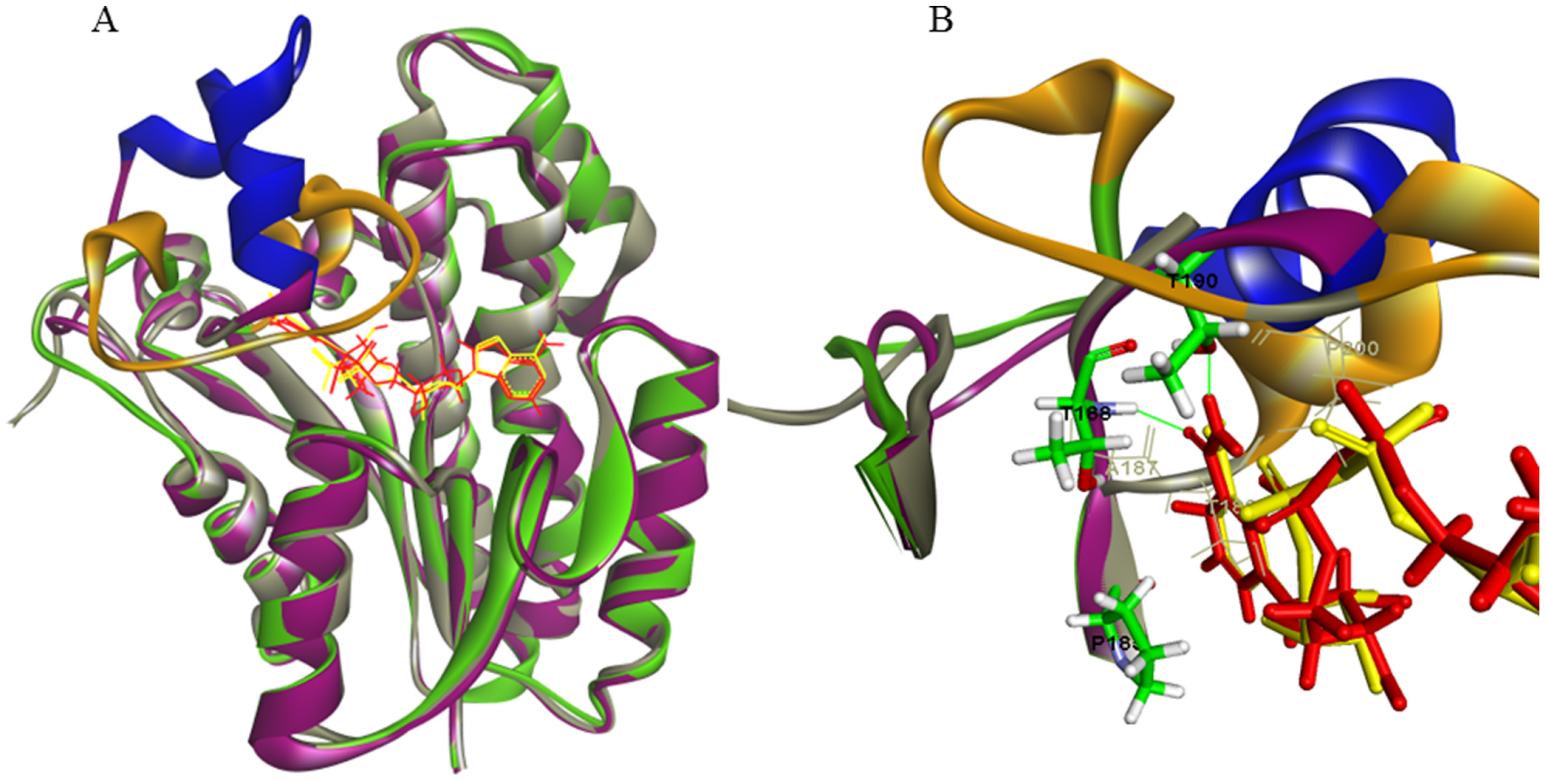
Superposition of the NAD^+^ bound binary complex (1fk8) with the molecular modeled structures of apo- and holoenzyme. (A) Ribbon diagram of the crystal structures of 3α-HSD/CR (grey) and the modeled structures of apo- (green) and holoenzyme (purple). The conformational changes in the inserted flexible substrate binding loops are shown in gold and blue for apo- and holoenzyme, respectively. NAD^+^ from the crystal structure and the modeled binary complex is shown as a line with yellow and red colors, respectively. (b) Close-up view of the potential steric hindrance in residues A187, T188, and P200 (grey line) of apoenzyme with NAD^+^ molecule in a holoenzyme model. Close contacts is not possible in the structure without causing conformational changes in the loop. T188 and T190 form hydrogen bonds (green line) with nicotinamide of NAD^+^ in the modeled holoenzyme.

The substrate-binding loop, which is not resolved in the crystal structure of 3α-HSD/CR, including the residues of T188-K208 and L192-K208 for apo- and holoenzyme, respectively, was modeled into the structure of the 3α-HSD/CR. A model of NAD^+^-bound complex was derived from a sequence alignment and is based on the crystal structure of *Pseudomonas sp.* 3α-HSD (PDB: 2DKN). The model of the substrate-binding loop of apoenzyme exhibits an open conformation with a loop of G186-T188, following a helix of E189-A195 and a loop of G196-E218. The binding of NAD^+^ with apoenzyme in the modeled structure changes the loop conformation and exhibits a closed form of a helix-turn-helix structure with a loop of G186-T190, a helix of P191-Q198, a turn of D199-Y202, a helix of G203-A207and then a loop of K208-E218 connected to the helix of the protein core structure (Figure 7). In the modeled holoenzyme, both T188 and T190 form hydrogen bonds with nicotinamide of NAD^+^ during the conformational change.

## Discussion

The functional roles of the fluctuating conformations and the dynamic motions of enzymes are demonstrated as being coupled with the binding and release of substrate or product and they facilitate the reaction. The conformational changes can be induced by binding with reactants and arrangement into a form suitable for the formation of a transition-state in an induced fit model of the enzyme-substrate interaction. It takes part in the enzyme specificity by acting as a molecular switch to slow release of the right substrate and fast dissociation of an incorrect substrate [33]. The enzyme may also exhibit fluctuating conformations to allow the sampling of ligand-bound conformations in the conformational selection model, where binding with ligands stabilizes the active conformer, thereby resulting in a population shift toward the active form [34]–[37]. The motion of the loop in the enzyme catalysis participates in binding with the ligands, interacting with the transition-state to facilitate the reaction and causing a release of the products. This study demonstrates that mutations in the flexible substrate-binding loop of 3α-HSD/CR affect the conformational change, the affinity with ligands and the kinetic parameters for catalysis, which results in a rate-limiting step in the catalysis for the hydrogen transfer in the central complexes.

The crystal structure of 3α-HSD/CR shows disorder in the substrate-binding loop of T188-K209. NAD^+^ bound with 3α-HSD/CR stabilizes the partial loop of the residues, T188-E189-T190-P191, in the binary complex where T188 is close to NAD^+^. In comparison with the structures of the NAD^+^-bound complex between *C.t.* 3α-HSD/CR and *P.s.* 3α-HSD, the unresolved region of L192-K208 in *C.t.* 3α-HSD/CR becomes a helix-turn-helix structure in *P.s.* 3α-HSD (Figure 2). The movement of the substrate-binding loop has been implicated in the binding with the ligand. Molecular modeling was performed to simulate the conformations of the substrate-binding loop in *C.t.* 3α-HSD/CR. The simulated loop appears to be exposed to the solvent, which is consistent with the fluorescence spectrum of the double mutants of W173F/P185W and W173F/T188W with a maximum wavelength at 345 and 349 nm, respectively (Figure 7). The residue, W188, in the loop is more exposed to the solvent than the residue, W185, in the hinge region. Molecular modeling shows that the loop containing residues, A187-E218, changes the conformation, when NAD^+^ bound in the active site closes the active site cleft. The conformational change requires a rearrangement of the flexible loop containing the residues, A187-P200, so that the coenzyme and some amino acid residues from the loop can be accommodated. There are steric conflicts in the residues of A187, T188 and P200 in apoenzyme with NAD^+^ bound in the closed form of the wild-type holoenzyme (Figure 7B). These steric conflicts are relieved by the movement of the substrate-binding loop. Therefore, this conformational change is necessary for substrate binding and appears to control the rate of dissociation of the coenzyme and the catalysis. This is discussed later.

The substrate-binding loop in SDR is usually located between the βF sheet and the αG helix and is flanked by two proline residues (P185 and P212 in 3α-HSD/CR) [20]. These two prolines may cause rigidity in the backbone and prevent conformational changes from propagating to the rest of the protein. The P185 in 3α-HSD/CR is situated at the hinge region of the loop and interacts with the hydroxyl group of S114. In addition, residue P185 is adjacent to the nicotinamide ring of NAD^+^ within a distance of 4.1 Å (Figure 2B). A NADH-induced secondary structure change of S114A mutant enzymes was detected (14). However, this conformational change leads to a nonproductive mode and causes a significant decrease in the value of *k_cat_*. Structural characterizations of the apo- and binary complex of wild-type and mutants with NADH were then conducted using circular dichroism and fluorescence spectroscopy. A change in the secondary structure for the enzyme variants is observed with mutation at P185. There is no difference for those variants with mutation at T188 compared to the wild-type enzyme. The cyclic side chain of proline prevents the rotation of an N-C_α_ bond, which results in structural rigidity in the backbone. The substitution of proline with a glycine or alanine relieves the rigidity of the backbone and increases the flexibility of the loop. This may result in a large change in the backbone conformation and there is an observed change in the overall secondary structure. No further induced change in the secondary structure by ligands was observed, when NADH and androsterone are added to the enzyme variants with mutation at P185 and T188 to form binary and ternary complexes. The change in the overall secondary structure caused by mutation on P185 seems to have no effect on the local environment of W173, since the same maximum wavelength at 329 nm in the intrinsic protein fluorescence emission of W173 for the wild-type and mutant enzymes is observed. However, the increased flexibility of the substrate-binding loop due to mutation at P185 relieves the quenching on the W173 fluorescence, giving a fluorescence intensity that increases in the order: P185G>P185A>WT.

### The Role of P185 and T188 in Substrate Binding

Mutation at either P185 or T188 results in a significant increase in the dissociation constant (*K_d_*) of the NADH-enzyme complex as determined by fluorescence titration at pH 7.5, and the inhibition constant (*K_iNAD_*) of NAD^+^-enzyme complex by steady-state kinetics at pH 10.5. The residue, P185, is important in maintaining the conformation of the substrate-binding loop. The substitution of P185 for alanine and glycine not only increases the local flexibility of this loop, but also interrupts any potential hydrophobic interaction with the nicotinamide ring and weakens the binding with the nucleotide cofactors of NADH and NAD^+^. Moreover the more hydrophobic substituted group seems to increase the interaction with both NAD^+^ and androsterone, and decreases the inhibition constant for NAD^+^ and the value of *K_m_* for androsterone in the order: WT<P185A<P185G. The distance between the hydroxyl group of the T188 residue and the nicotinamide of NAD^+^ in the crystal structure is 3.6 Å. The replacement of T188 with alanine interrupts the potential hydrogen bonding interaction between the nicotinamide of NAD(H) with the hydroxyl group of threonine, leading to a severe weakening of the binding of the nucleotide cofactor, thereby increasing the value of *K_d_* and *K_iNAD_* for the binary complex of the NADH-T188A mutant and the NAD^+^-T188A mutant, respectively, in comparison with the value for the wild-type enzyme. Residue T188 is also important to binding with androsterone. Mutation causes a 14-fold increase in the value of *K_androsterone_*. Unexpectedly, the replacement of T188 by serine has a similar effect on the kinetic parameters with T188A mutant enzyme and does not restore the binding constant for NAD^+^. This result is not predictable since the hydroxyl group of serine is capable of interacting with the amide group of nicotinamide and suggests that both the hydroxyl group and the methyl group in the side chain of T188 play an important role in hydrogen bonding and in maintaining the orientation for the interaction with NAD(H). Therefore, the rigidity of P185 at the hinge region is important in maintaining the loop conformation to allow interaction with NAD(H), while the hydroxyl group of T188 is important in hydrogen bonding with the amide group of nicotinamide.

### The Role of P185 and T188 in Catalysis

3α-HSD/CR catalyzes the oxidation of androsterone with NAD^+^ to form androstanedione and NADH. A previous study by the authors demonstrated the kinetic isotope effects of *^D^V_max_* of 1, *^D^*(*V_max_/K_androsterone_*) of 1.8, and *^D2O^V_max_* of 2.1, suggesting that the release of the product NADH is coupled with proton transfer and is the rate-limiting step in the overall reaction catalyzed by wild-type 3α-HSD/CR, thereby masking the kinetic isotope effect in the chemical step [4]. The rate-limiting step, which occurs on release of the product, is also coupled with the conformational change of the enzyme [38], [39]. In order to measure the rate constants for the 3α-HSD/CR catalyzed reaction, stopped-flow experiments were performed. However, the rate constant for the hydride transfer in the enzyme-catalyzed reaction is too fast to be detected in the dead time of the stopped-flow measurement. Therefore, a stereospecific labeled [3α-^2^H] androsterone was used to slow the step for the hydride transfer. A burst phase accompanies the exponential increase, followed by a linear increase in the formation of the deuterated NADH, giving an apparent rate constant of 309 s^−1^ for the deuteride transfer. Because the observed ^D^(*V_max_/K_androsterone_*) value is 1.8, the minimum rate constant of 556 s^−1^ is calculated for hydride transfer from the undeuterated androsterone to NAD^+^. In addition, the observed burst phase is consistent with the rate-limiting step for NADH release.

The function of P185 and T188 residues was then characterized by a steady-state kinetic study of the oxidation of androsterone with NAD^+^ at pH 10.5. Firstly, we assessed the rate-limiting step by studying the primary isotope effect on the hydride transfer. The kinetic parameter, *V_max_*, combines all of the steps from the ternary complex of 3α-HSD/CR-NAD^+^-androsterone to the release of the last product (NADH) in the reaction, while *V_max_/K_androsterone_* includes the steps from the binding of androsterone to the first irreversible step, *i.e.,* the release of androstanedione. The size of deuterium kinetic isotope effect reflects hydride transfer step. An equal primary kinetic isotope effect on *V_max_* and *V_max_/K_androsterone_* may result from the rate-limiting step on the hydride transfer. Any rate-limiting steps outside the hydride transfer step mask the intrinsic kinetic isotope effect, resulting in an observed isotope effect that is close to 1 [40]. A finite equal primary deuterium isotope effect of around 1.8 on *V_max_* and *V_max_/K_androsterone_* is observed for the mutants of P185A, P185G, T188A and T188S. This result suggests that mutation at either P185 or T188 changes the rate-limiting step for the hydride transfer and so product release is not a rate-limiting step for the mutants of P185A, P185G, T188A and T188S. Either slowing the chemical step or increasing the dissociation rate of NADH produces the rate-limiting step for the hydride transfer. Secondly, the kinetic mechanisms for the mutated enzymes were studied. The initial rate patterns indicate steady-state sequential kinetics for P185A and P185G mutant enzymes, while rapid equilibrium sequential kinetics are observed for the T188A and T188S mutant enzymes. This suggests that the release of NAD^+^ is fast compared to the chemical step for the hydride transfer for T188A and T188S mutant enzymes [41]. Therefore, based on a study of the kinetic isotope effects and the initial rate patterns, the rate-limiting step is the release of NADH for the oxidation of androsterone with NAD^+^ catalyzed by the wild-type enzyme, and changes to hydride transfer with the T188A and T188S mutant enzymes. The catalytic constant (*k_cat_*) is increased 3–14-fold for the P185A, P185G, T188A and T188S mutant enzymes, compared to the value for the wild-type enzyme. The increase in the catalytic constant further confirms that mutation causes an increase in the rate of product release, thereby increasing the overall rate constant. However, the catalytic efficiency (*k_cat_/K_NAD_*) for NAD^+^ decreases in the order: WT>P185A>P185G, which suggests that the increased flexibility of the substrate binding loop reduces the catalytic efficiency of NAD^+^, possibly due to the increased entropy of the loop [24]. An increase in the flexibility of the polypeptide backbone may result in a decreased probability of the formation of a catalytically productive active site and also a decrease in the interaction between the substrate-binding loop with NAD(H).

**Figure 8:**
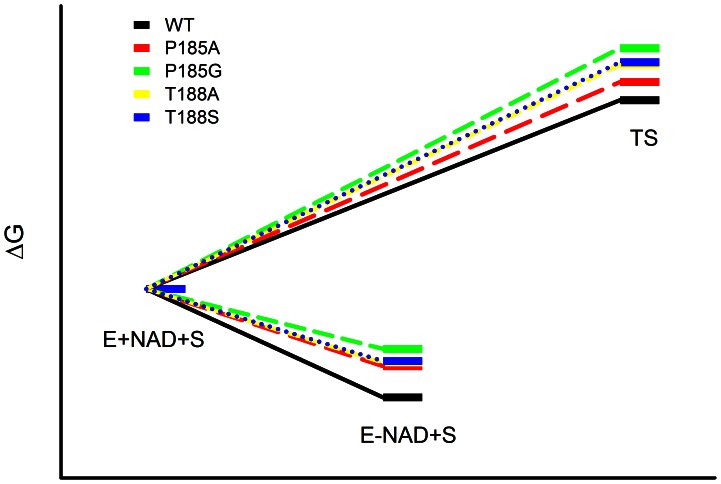
Free energy profiles for catalysis by wild-type and mutant 3α-HSD/CRs. The free energy differences calculated from the data in Table 2 are normalized to the free energy of the ground state (E+NAD+S), containing the enzyme of wild-type and mutants (E), and the free substrates, NAD^+^ and androsterone (S). Energy levels are shown for the ground binding energy of the enzyme-NAD^+^ complex (E-NAD+S), and the transition state (TS) energy for the hydride transfer from androsterone to NAD^+^ in the reaction catalyzed by the wild-type and mutant 3α-HSD/CRs, respectively. The free energy profiles illustrate the differential destabilization of the ground state and the transition state caused by the mutation at P185 and T188 in the 3α-HSD/CR catalyzed reaction, as described in the text.

The effect of each mutation on the binding interactions between the enzyme and the nucleotide cofactor NAD^+^ were evaluated using the value of *K_iNAD_*. The transition state of hydride transfer from enzyme-bound NAD^+^ to androsterone was evaluated using the kinetic parameters *k_cat_/K_iNAD_K_androsterone_* (Figure 8). The rate constant of *k_cat_/K_iNAD_K_androsterone_* provides a measure of the energy barrier for the conversion of androsterone and NAD^+^ to the transition state for the reaction catalyzed by 3α-HSD/CR. The value of *K_iNAD_* is 0.06 mM for the binary complex of NAD^+^ with wild-type 3α-HSD/CR at pH 10.5, with increases of 23- and 27-fold for the T188A and T188S mutant enzymes, respectively. This gives a ground state destabilization (ΔΔ*G_b mut/wt_*) of 1.8 and 1.9 kcal/mol for the NAD^+^ bound with T188A and T188S mutant enzymes, respectively. Mutation at T188A and T188S destabilizes the transition state (ΔΔ*G* ^‡^ *_mut/wt_*) for the oxidation of androsterone with NAD^+^ by 1.9 and 2.0 kcal/mol, respectively. Therefore, T188 residue in the substrate-binding loop contributes to a similar stabilization of the transition state and the ground state. Therefore, mutation at residue T188 increases the off rate of NAD^+^ without a sacrifice of the activation energy to facilitate the hydride transfer. The value of *K_iNAD_* for P185A and P185G mutants is increased 17- and 83-fold, respectively. This gives a ground-state destabilization of 1.7 and 2.6 kcal/mol for the NAD^+^ bound with P185A and P185G mutants, respectively. The effect of each mutation on the destabilization of the transition state was then calculated for P185A and P185G mutants, giving 0.96 and 2.8 kcal/mol, respectively. The substitution of proline into glycine causes a similar degree of destabilization of both the ground-state and the transition-state. Mutation at residue P185 increases the flexibility of the substrate-binding loop, similarly affecting the binding interaction with both the ground-state and the transition-state. Meanwhile a more hydrophobic substituted group, such as alanine, favors binding with NAD^+^.

In conclusion, the substrate-binding loop of 3α-HSD/CR plays an important role in the binding of the nucleotide cofactor and androsterone and assists catalysis and facilitates product release. 3α-HSD/CR from *C. testosteroni* reduces the activation energy of the hydride transfer during catalysis, resulting in a rate-limiting step for the product release. A balance of structural rigidity and flexibility for the substrate-binding loop is essential for enzyme catalysis. The substrate-binding loop in the apoenzyme is exposed to the solvent and its flexibility allows access to the substrate. Binding of the nucleotide cofactor may induce the closed form, which is stabilized by hydrogen bonding between the amide NH of the nicotinamide ring and the hydroxyl group of T188 on the loop and the hydrophobic interaction of the nicotinamide ring with the proline ring in the hinge region. The rigid P185 at the hinge region and the hydrogen bonding between the nucleotide cofactor and T188 favor the formation of the closed conformation to allow arrangement into a form suitable for the formation of a transition-state. Either relieving the strain by the mutation at P185 or interrupting the hydrogen bonding by mutation at T188 destabilizes the transition state for the hydride transfer, but increases the flexibility of the substrate-binding loop, thereby increasing the rate of product release and the overall activity. This study determines the role of the flexibility of the substrate-binding loop and provides an insight into the conformational changes and catalysis in the related SDR superfamily enzymes.

## Supporting Information

Figure S1 **Initial rate pattern for the mutated 3α-HSD/CRs.** The initial rate pattern was obtained by varying the concentration of androsterone at several fixed concentrations of NAD^+^ in 0.1 M Caps at pH 10.5. The lines represent the fit of data points to Equation 2 for the P185A, and P185G mutant enzymes, while data were fitted to Equation 3 for the T188A and T188S mutant enzymes.
(TIF)

Figure S2 **Isotope effects on 3α-HSD/CR catalyzed reaction.** The reactions were performed with varying concentrations of the deuterated and unlabeled androsterone at 1.7 mM NAD^+^, pH 10.5. The lines represent the fit of data points to Equation 7 for the P185A, P185G, T188A and T188S mutant enzymes. The kinetic parameters of wild-type enzyme are from Ref. 4 and fit the data points to Equation 6.
(TIF)

Table S1 **Oligonucleotide primers used for site-directed mutagenesis.**
(DOCX)
