# Supplementary figures and images for: The Catalytic Roles of P185 and T188 and Substrate-Binding Loop Flexibility in 3α-Hydroxysteroid Dehydrogenase/Carbonyl Reductase from Comamonas testosteroni
Source: PLoS One. 2013 May 23;8(5):e63594. doi: 10.1371/journal.pone.0063594 (PMC3662788; doi:10.1371/journal.pone.0063594)

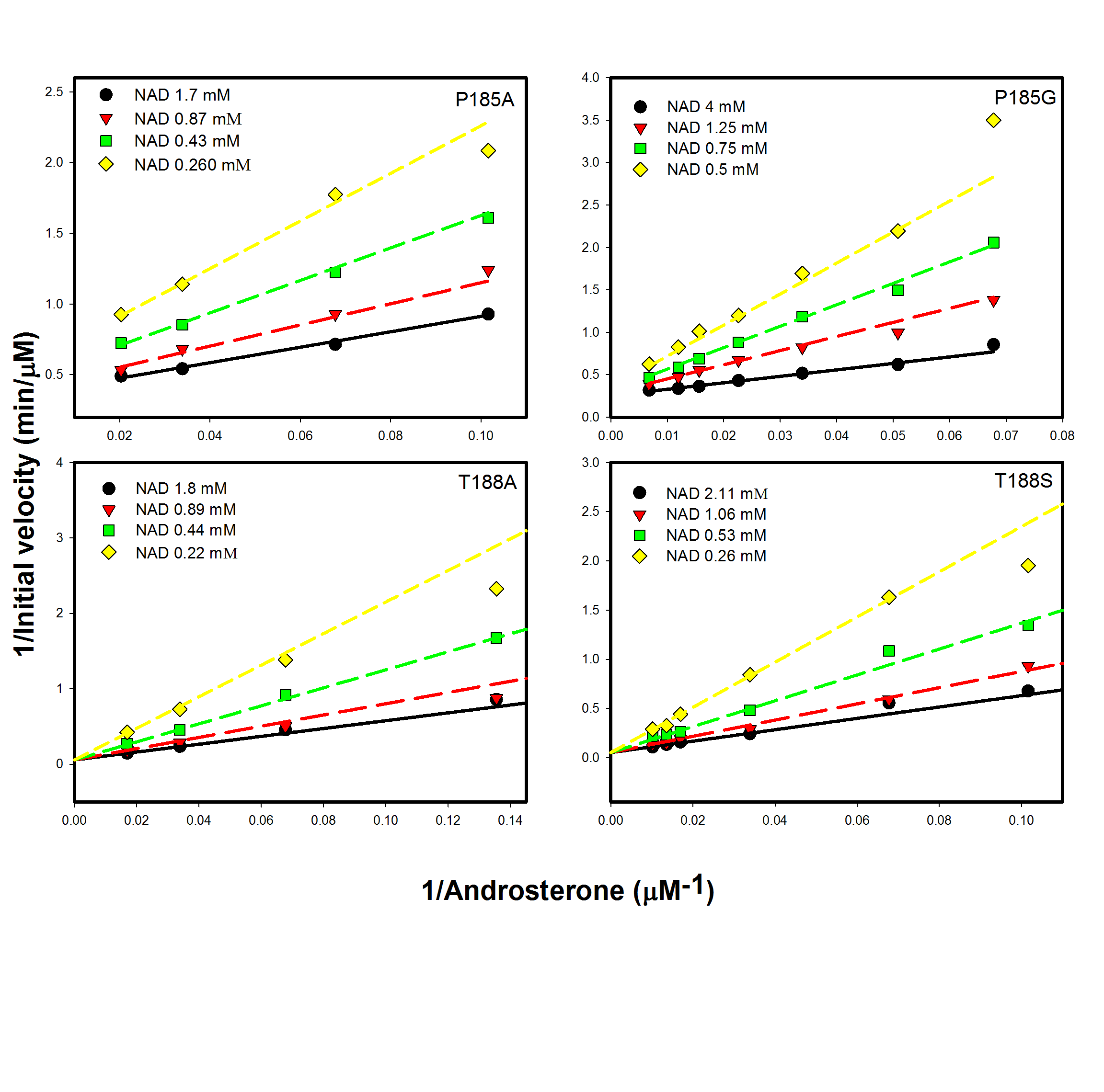

Supplement: Figure S1 — Initial rate pattern for the mutated 3α-HSD/CRs. The initial rate pattern was obtained by varying the concentration of androsterone at several fixed concentrations of NAD+ in 0.1 M Caps at pH 10.5. The lines represent the fit of data points to Equation 2 for the P185A, and P185G mutant enzymes, while data were fitted to Equation 3 for the T188A and T188S mutant enzymes. (TIF) [file pone.0063594.s001.tif]

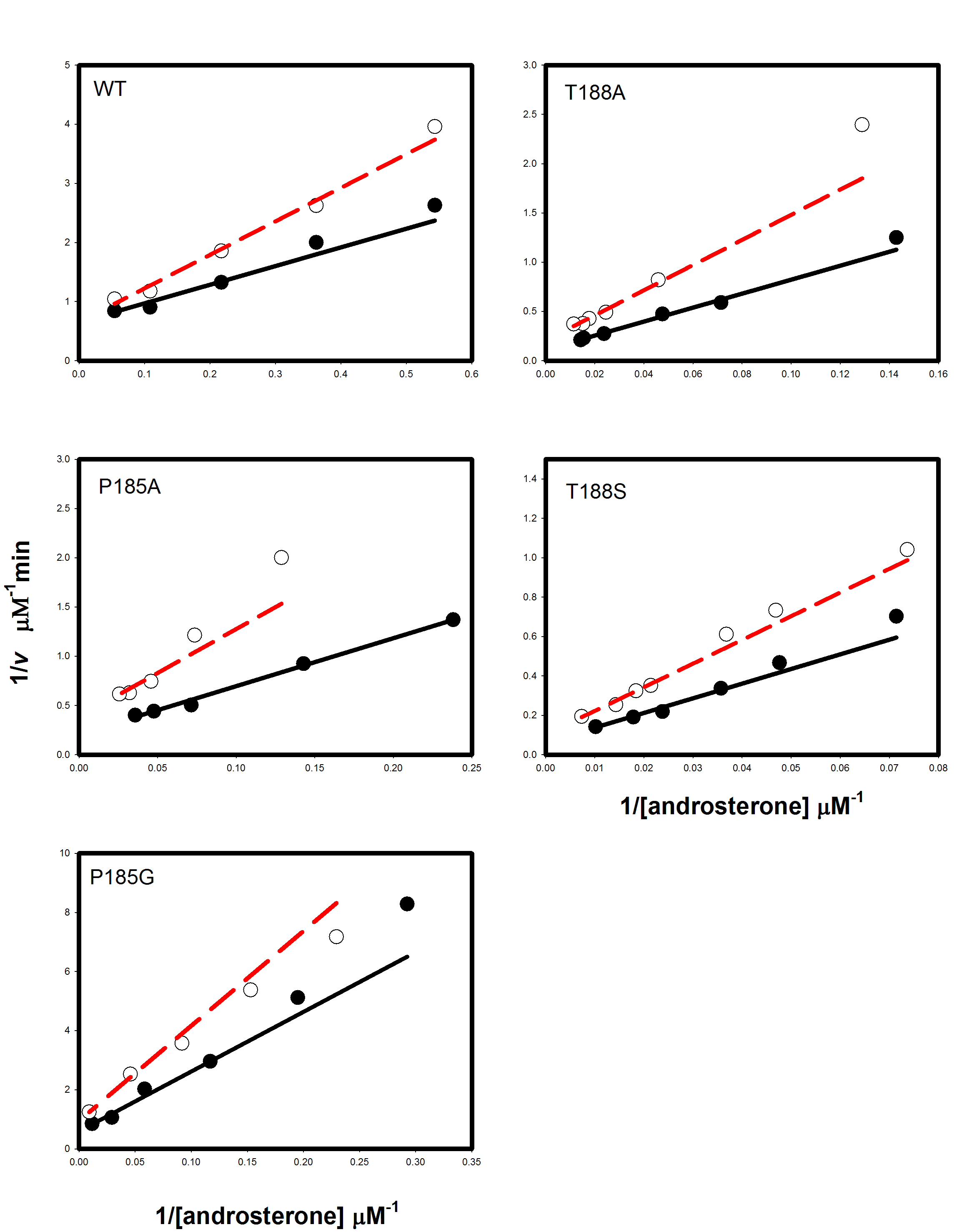

Supplement: Figure S2 — Isotope effects on 3α-HSD/CR catalyzed reaction. The reactions were performed with varying concentrations of the deuterated and unlabeled androsterone at 1.7 mM NAD+, pH 10.5. The lines represent the fit of data points to Equation 7 for the P185A, P185G, T188A and T188S mutant enzymes. The kinetic parameters of wild-type enzyme are from Ref. 4 and fit the data points to Equation 6. (TIF) [file pone.0063594.s002.tif]
