# Supplementary material for: The Catalytic Roles of P185 and T188 and Substrate-Binding Loop Flexibility in 3α-Hydroxysteroid Dehydrogenase/Carbonyl Reductase from Comamonas testosteroni
Source: PLoS One. 2013 May 23;8(5):e63594. doi: 10.1371/journal.pone.0063594 (PMC3662788; doi:10.1371/journal.pone.0063594)
Supplement: Table S1 — Oligonucleotide primers used for site-directed mutagenesis. (DOCX) [file pone.0063594.s003.docx]

**Table S1. Oligonucleotide primers used for site-directed mutagenesis^a^**

| Mutation | Direction | Nucleotide sequence of primer^b^ |
| --- | --- | --- |
| P185A | Forward | 5'- ctgaacaccatcgcc**gcc**ggtgcaaccgagactccc-3' |
|  | Reverse | 5'- gggagtctcggttgcacc**ggc**ggcgatggtgttcag-3' |
| P185G | Forward | 5'- ctgaacaccatcgcc**ggc**ggtgcaaccgagactccc-3' |
|  | Reverse | 5'- gggagtctcggttgcacc**gcc**ggcgatggtgttcag-3' |
| P185W | Forward | 5'- ctgaacaccatcgcc**tgg**ggtgcaaccgagactccc-3' |
|  | Reverse | 5'- ctgaacaccatcgcc**cca**ggtgcaaccgagactccc-3' |
| T188A | Forward | 5'- ccatcgcccccggtgca**gcc**gagactcccttgctgcag-3' |
|  | Reverse | 5'- ctgcagcaagggagtctc**ggc**tgcaccgggggcgatgg-3' |
| T188S | Forward | 5'- ccatcgcccccggtgca**tcc**gagactcccttgctgcag-3' |
|  | Reverse | 5'- ctgcagcaagggagtctc**gga**tgcaccgggggcgatgg-3' |
| T188W | Forward | 5´-ccatcgcccccggtgca**tgg**gagactcccttgctgcag-3´ |
|  | Reverse | 5´-ctgcagcaagggagtctc**cca**tgcaccgggggcgatgg-3´ |
| W173F | Forward | 5´-aaacgcgccgccgcc**ttc**ggcgaggctggcgtgcgc-3´ |
|  | Reverse | 5´-gcgcacgccagcctcgcc**gaa**ggcggcggcgcgttt-3´ |

^a^ The plasmid for double mutants of T188W/W173F and P185W/W173F were constructed with W173F plasmid with T188W and P185W primers, respectively. ^b^ The boldface codons indicate the mutation on the amino acid residues.
